# Performance of the Swiss Digital Contact-Tracing App Over Various SARS-CoV-2 Pandemic Waves: Repeated Cross-sectional Analyses

**DOI:** 10.2196/41004

**Published:** 2022-11-11

**Authors:** Paola Daniore, Vasileios Nittas, Tala Ballouz, Dominik Menges, André Moser, Marc Höglinger, Petra Villiger, Krisztina Schmitz-Grosz, Viktor Von Wyl

**Affiliations:** 1 Institute for Implementation Science in Healthcare University of Zurich Zurich Switzerland; 2 Digital Society Initiative University of Zurich Zurich Switzerland; 3 Epidemiology, Biostatistics and Prevention Institute University of Zurich Zurich Switzerland; 4 Clinical Trials Unit University of Bern Bern Switzerland; 5 Winterthur Institute of Health Economics Zurich University of Applied Sciences Winterthur Switzerland; 6 Medgate Aktiengesellschaft Basel Switzerland

**Keywords:** digital contact tracing, exposure notification, COVID-19, SARS-CoV-2, public health, surveillance, digital proximity, contact-tracing app, mobile app, Switzerland, variant of concern, SwissCovid app, digital tool

## Abstract

**Background:**

Digital proximity-tracing apps have been deployed in multiple countries to assist with SARS-CoV-2 pandemic mitigation efforts. However, it is unclear how their performance and effectiveness were affected by changing pandemic contexts and new viral variants of concern.

**Objective:**

The aim of this study is to bridge these knowledge gaps through a countrywide digital proximity-tracing app effectiveness assessment, as guided by the World Health Organization/European Center for Prevention and Disease Control (WHO/ECDC) indicator framework to evaluate the public health effectiveness of digital proximity-tracing solutions.

**Methods:**

We performed a descriptive analysis of the digital proximity-tracing app SwissCovid in Switzerland for 3 different periods where different SARS-CoV-2 variants of concern (ie, Alpha, Delta, and Omicron, respectively) were most prevalent. In our study, we refer to the indicator framework for the evaluation of public health effectiveness of digital proximity-tracing apps of the WHO/ECDC. We applied this framework to compare the performance and effectiveness indicators of the SwissCovid app.

**Results:**

Average daily registered SARS-CoV-2 case rates during our assessment period from January 25, 2021, to March 19, 2022, were 20 (Alpha), 54 (Delta), and 350 (Omicron) per 100,000 inhabitants. The percentages of overall entered authentication codes from positive tests into the SwissCovid app were 9.9% (20,273/204,741), 3.9% (14,372/365,846), and 4.6% (72,324/1,581,506) during the Alpha, Delta, and Omicron variant phases, respectively. Following receipt of an exposure notification from the SwissCovid app, 58% (37/64, Alpha), 44% (7/16, Delta), and 73% (27/37, Omicron) of app users sought testing or performed self-tests. Test positivity among these exposure-notified individuals was 19% (7/37) in the Alpha variant phase, 29% (2/7) in the Delta variant phase, and 41% (11/27) in the Omicron variant phase compared to 6.1% (228,103/3,755,205), 12% (413,685/3,443,364), and 41.7% (1,784,951/4,285,549) in the general population, respectively. In addition, 31% (20/64, Alpha), 19% (3/16, Delta), and 30% (11/37, Omicron) of exposure-notified app users reported receiving mandatory quarantine orders by manual contact tracing or through a recommendation by a health care professional.

**Conclusions:**

In constantly evolving pandemic contexts, the effectiveness of digital proximity-tracing apps in contributing to mitigating pandemic spread should be reviewed regularly and adapted based on changing requirements. The WHO/ECDC framework allowed us to assess relevant domains of digital proximity tracing in a holistic and systematic approach. Although the Swisscovid app mostly worked, as reasonably expected, our analysis revealed room for optimizations and further performance improvements. Future implementation of digital proximity-tracing apps should place more emphasis on social, psychological, and organizational aspects to reduce bottlenecks and facilitate their use in pandemic contexts.

## Introduction

To contribute to mitigation efforts against the spread of SARS-CoV-2, digital proximity-tracing apps were developed and widely adopted in multiple countries. This gave rise to a novel research area within digital public health, which aims to assess the possible contribution of such apps toward disease control. Prominent examples of digital proximity-tracing apps in Europe include the United Kingdom’s National Health Service’s (NHS) COVID-19 app, the German Corona-Warn-App, and the SwissCovid app from Switzerland [1-3]. In Switzerland, smartphone ownership exceeding 90% [4] across all socioeconomic groups presented an opportunity for the SwissCovid app to be widely adopted and complement manual contact-tracing efforts. Conducted in the form of interviews, manual contact tracing is labor intensive and prone to errors due to its reliance on people’s abilities to recall proximity contacts [5]. The SwissCovid app promised to deliver exposure notifications at a faster rate, with broader reach and greater scalability [6,7]. However, it was essential that exposure notifications be sent quickly and without interruptions, ultimately providing a time advantage over manual contact tracing [8].

There is growing interest in further evaluating the effectiveness of digital proximity-tracing apps. However, effectiveness analyses face multiple challenges [7,9]. First, the outcome of interest, which is the prevention of SARS-CoV-2 transmission, is not observable. Second, the privacy-preserving architecture of digital proximity-tracing apps, particularly those that follow the Decentralized Privacy-Preserving Proximity Tracing (DP-3T) blueprint [10], provides only limited, nonidentifiable data for conducting effectiveness analyses. Lastly, additional relevant data generated, for example, through manual contact tracing, information hotlines, and testing centers, henceforth described as “points of contact for app users,” are often dispersed across different systems and not readily available due to privacy regulations [11].

Empirical evaluations of the effectiveness of digital proximity-tracing apps remain scarce [12]. Recent evaluations have mainly produced mixed results, ranging from substantial [13-15] to moderate [16,17] or disappointing [18] findings. There is also a large heterogeneity of analytical methods and data used for these analyses, which makes a direct comparison of their results difficult. To foster standardization, the World Health Organization (WHO) and the European Center for Disease Prevention and Control (ECDC) recently developed a framework outlining the most relevant data and monitoring indicators for digital contact-tracing apps (henceforth referred to as the “WHO/ECDC framework”) [19]. To the best of our knowledge, however, this framework has not yet been applied to a systematic, countrywide analysis, and its utility for effectiveness analyses remains to be explored.

The aim of this study is to bridge these knowledge gaps through a countrywide digital proximity-tracing app effectiveness assessment, as guided by the WHO/ECDC framework. Specifically, we performed a descriptive analysis of the digital proximity-tracing app in Switzerland for 3 different periods where different SARS-CoV-2 variants of concern (ie, Alpha, Delta, and Omicron, respectively) were most prevalent. We performed this analysis by applying the WHO/ECDC framework to individual and public-level data, which we complemented with additional indicators of mitigative actions taken by app users after receiving an exposure notification. Accordingly, our analysis applies the WHO/ECDC framework indicators in the greater pandemic context to inform future indicator-based app monitoring and effectiveness assessment efforts.

## Methods

### SwissCovid Digital Proximity-Tracing App

Switzerland was 1 of the first countries that launched a digital proximity-tracing app (SwissCovid) based on the DP-3T architecture on June 25, 2020 [20]. The DP-3T architecture works by sending low-energy Bluetooth beacons with a pseudonymized, regularly changing user identification number to other SwissCovid app users in its surroundings. Here, the Bluetooth signal strength serves as a proxy for the physical distance between 2 smartphones. Copies of a user’s own identification numbers, as well as those of recent proximity encounters with other apps, are then stored locally on the users’ smartphones.

The SwissCovid app worked through an exposure notification cascade system to identify and isolate possible SARS-CoV-2 cases of interest. The exposure notification cascade started when a user received a positive polymerase chain reaction (PCR) test result for SARS-CoV-2. This triggered the first step in the cascade (illustrated in Supplementary Figure 1 in Multimedia Appendix 1), in which the user was issued an authentication code. Users subsequently entered their authentication code in the app, leading to the release of their own pseudonymized identification numbers to a central server. The SwissCovid app regularly downloaded identification numbers and searched locally registered identification numbers from proximity encounters. An exposure notification was triggered by the app if contact exposure between 2 or more individuals met predefined proximity and time thresholds (proximity of ≤1.5 m to an infected person for ≥15 minutes). This message included further instructions for the exposed individuals, such as the phone number for a SwissCovid infoline and a link to a risk self-assessment web form (from December 2020). Exposure-notified SwissCovid app users were advised to call the infoline number and to seek free-of-charge SARS-CoV-2 testing.

During its operational period, until its deactivation on April 1, 2022, the SwissCovid app reached approximately 1.9 million users, corresponding to 26.1% of all Swiss inhabitants aged 16 years and older [20]. In total, 205,000 positive test results triggered exposure notifications through the SwissCovid app, and 141,000 infoline calls or web forms were completed. Further details on how digital proximity-tracing apps work [11] as well as existing evidence of SwissCovid app effectiveness in pandemic mitigation for Switzerland have been presented in detail elsewhere [17].

### Data Collection

Our study’s approach was guided by the WHO/ECDC framework. In brief, this framework provides a set of key indicators to guide the monitoring and evaluation of digital proximity-tracing apps, as well as to measure the performance and effectiveness of the corresponding exposure notification cascade in preventing onward transmission of SARS-CoV-2 (see Supplementary Table 1 in Multimedia Appendix 2).

We used data from public and nonpublic sources. Public monitoring data for the SwissCovid app [20] and the SARS-CoV-2 pandemic [21] were retrieved from the website of the Swiss Federal Office of Public Health. Data on the Oxford measurement of stringency of COVID-19 measures were retrieved from the respective website [22]. We also used data provided by the company that operated SwissCovid Infoline (Medgate Aktiengesellschaft) for aggregated daily counts of generated upload authentication codes, infoline calls, and self-assessment web entries. Additionally, we used longitudinal individual-level data, collected through surveys within the COVID-19 Social Monitor study, to provide additional indicators of interest regarding the mitigative actions taken by individuals upon receiving an exposure notification [23]. Further details on indicator definitions and data sources are presented in Multimedia Appendix 3.

### Statistical Analysis

Longitudinal analyses of SARS-CoV-2–monitoring indicators, defined in Supplementary Table 1 in Multimedia Appendix 2, were conducted for the entire study period from January 25, 2021, to March 19, 2022. Daily count values were averaged over 7 days or over the entire study period. Comparisons of SwissCovid app effectiveness indicators were conducted for stratified periods based on the 3 predominant SARS-CoV-2 variants of concern [21] and were aligned with the COVID-19 Social Monitor survey data collection phases: (1) Alpha variant (January 25-June 17, 2021, survey waves 13-17), (2) Delta variant (August 30-December 16, 2021, survey waves 18-20), and (3) Omicron BA.1 variant (January 24-March 19, 2022, survey waves 21-22); see Supplementary Figure 2 in Multimedia Appendix 4.

Our analysis focused on 3 of the WHO/ECDC framework indicators: (a) adoption of the SwissCovid app and frequency of exposure notifications, (b) successfulness of digital proximity-tracing apps in detecting contacts at risk of infection, and (c) whether digital proximity-tracing apps are faster in notifying contacts than conventional contact tracing. Specifically, all assessments in our analyses are linked to SwissCovid app users in their individual uptake and engagement with the app. The indicators further assess the performance and effectiveness of the SwissCovid app in mitigating onward viral transmission based on user responses to exposure notifications (ie, in forms of mitigative actions or noncompliance). To further provide context to the development of the indicators assessed in this study, we retrieved Oxford stringency index values for Switzerland, which quantify the strictness of countrywide lockdown policies during the SARS-CoV-2 pandemic [22].

To evaluate possible gaps in compliance with recommended measures, we defined a theoretical upper ceiling estimate for app users testing positive for SARS-CoV-2 infection. This upper ceiling estimate was calculated as the number of individuals who tested positive multiplied by the percentage of app users in the general population. Additional indicators were calculated based on mitigative actions taken by SwissCovid app users and by using individual-level data from the COVID-19 Social Monitor: (1) having been tested for SARS-CoV-2, (2) having tested positive for SARS-CoV-2, (3) having been in isolation or in quarantine ordered by a physician or manual contact tracing, and (4) having received an exposure notification (see Supplementary Figure 3 and Supplementary Table 4 in Multimedia Appendix 5).

Analyses were performed in Stata version 16.1 (StataCorp LLC). All data were analyzed descriptively as counts and percentages. Selected indicators were visualized using 3 topical radar plots. Reporting was informed by the Strengthening the Reporting of Observational Studies in Epidemiology (STROBE) checklist (Multimedia Appendix 6) [24].

### Ethical Considerations

For the COVID-19 Social Monitor study, the Cantonal Ethics Commission of Zurich concluded that our study did not fall within the scope of the Human Research Act (BASEC-Nr. Req-2020-00323). All other data did not require ethics approval.

## Results

### Longitudinal Analysis of Monitoring Indicators From Official Public Health Sources

**Figure 1:**
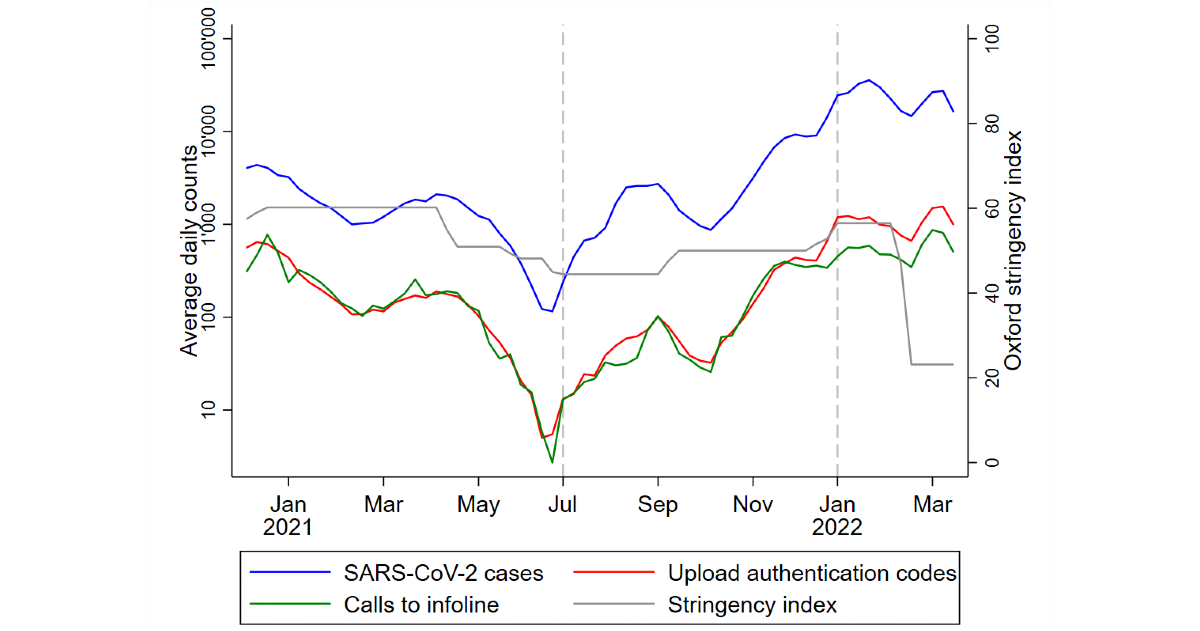
Longitudinal description of key indicators (7-day averages). The dashed vertical lines delineate different pandemic phases that were dominated by the Alpha, Delta, or Omicron SARS-CoV-2 variants of concern.

Figure 1 depicts the evolution of measured indicators across the 3 pandemic waves of the SARS-CoV-2 variants of concern. The blue line represents the counts of positive SARS-CoV-2 tests in Switzerland. The trend here suggests several incidence peaks in January 2021, which were due to the Alpha variant, and January 2022, which marks the transition of predominance from the Delta to the Omicron variant. The average daily cases over the study period were 20 (Alpha), 54 (Delta), and 350 (Omicron) per 100,000 inhabitants. The gray line illustrates the Oxford measure of stringency of COVID-19 measures, which ranges from 0 (lowest stringency) to 100 (highest stringency). In our observation period, the stringency of measures was highest between January and April 2021. This coincided with the Alpha variant phase, where measures such as home office and prohibition of gatherings were mandated by the Swiss Federal Office of Public Health. The stringency measure was also high during the final Delta variant phase and the beginning of the Omicron variant phase. Almost all mitigation measures were removed in February 2022.

The red and green lines illustrate the number of entered authentication codes by SARS-CoV-2–positive SwissCovid app users and calls to the infoline or completion of a self-assessment form upon receipt of an exposure notification, respectively. In the assessed period, the counts of these user-driven actions closely followed the incidence curve. Furthermore, they occurred in an almost stable 1:1 ratio, with 1 infoline call or completed web form per shared positive test result for the majority of the study period. However, there was a shift in this ratio deviating toward fewer user actions taken by exposed contacts during the Omicron variant phase.

### Indicator Comparisons Across Pandemic Phases

#### Indicators of Exposure Notification Cascade Performance

**Figure 2:**
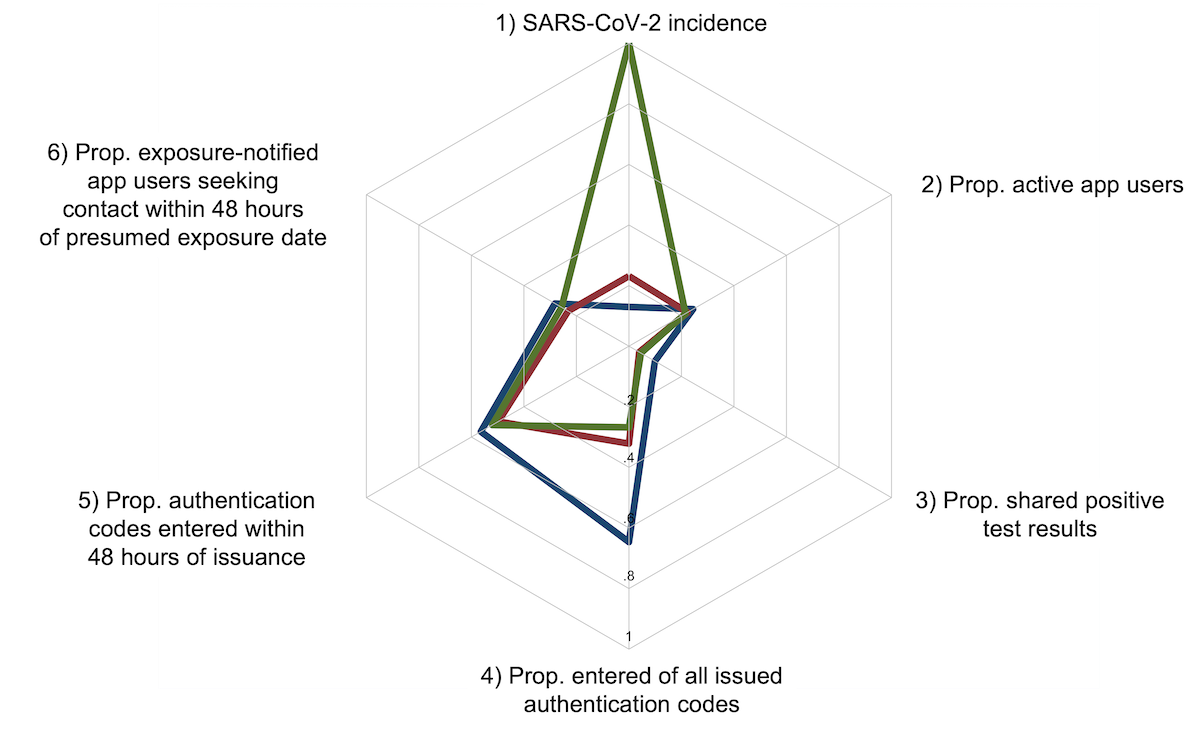
Indicators reflecting the performance of the exposure notification cascade. The colored lines represent the Alpha (blue), Delta (red), and Omicron (green) variant phases. The plot ranges from 0 (center) to 1 and illustrates the proportions and ratios of the relevant indicators. Indicator definitions and data sources are provided in Supplementary Table 1 in Multimedia Appendix 2. Prop.: proportion.

**Figure 3:**
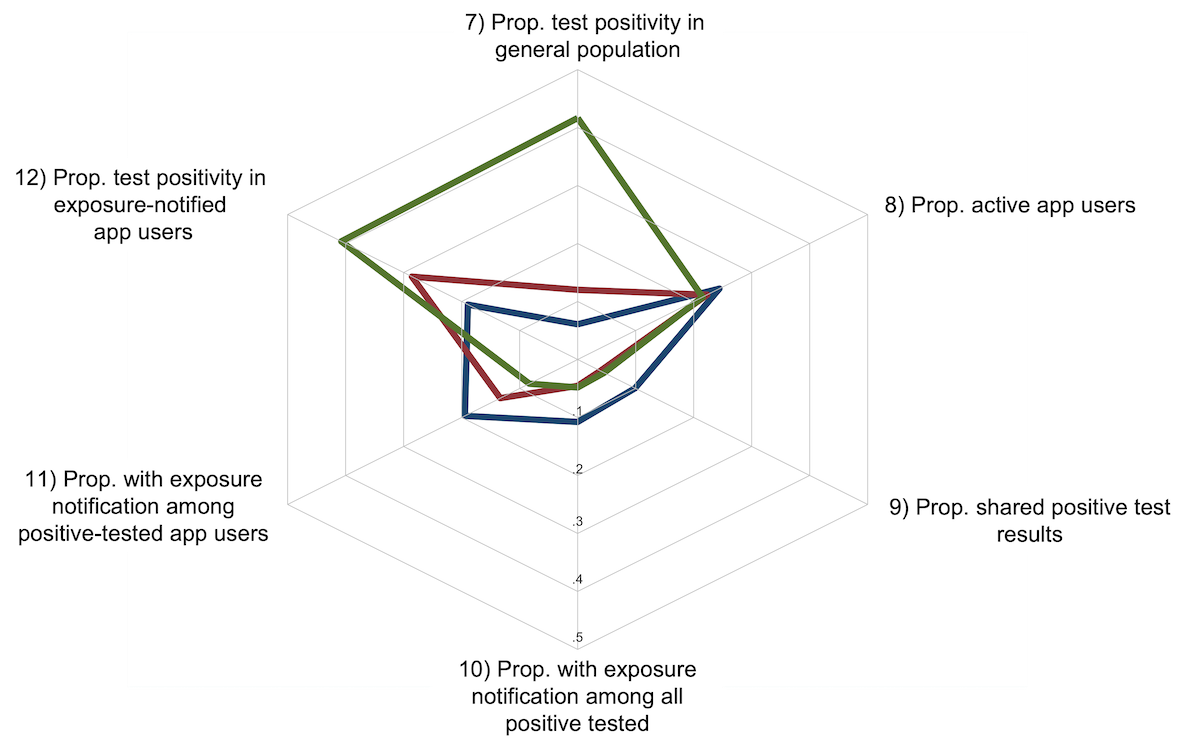
Indicators reflecting the proportion of exposure notifications or individuals who tested positive. The colored lines represent the Alpha (blue), Delta (red), and Omicron (green) variant phases. The plot ranges from 0 (center) to 0.5 and illustrates the proportions and ratios of the relevant indicators. Indicator definitions and data sources are provided in Supplementary Table 1 in Multimedia Appendix 2. Prop.: proportion.

**Figure 4:**
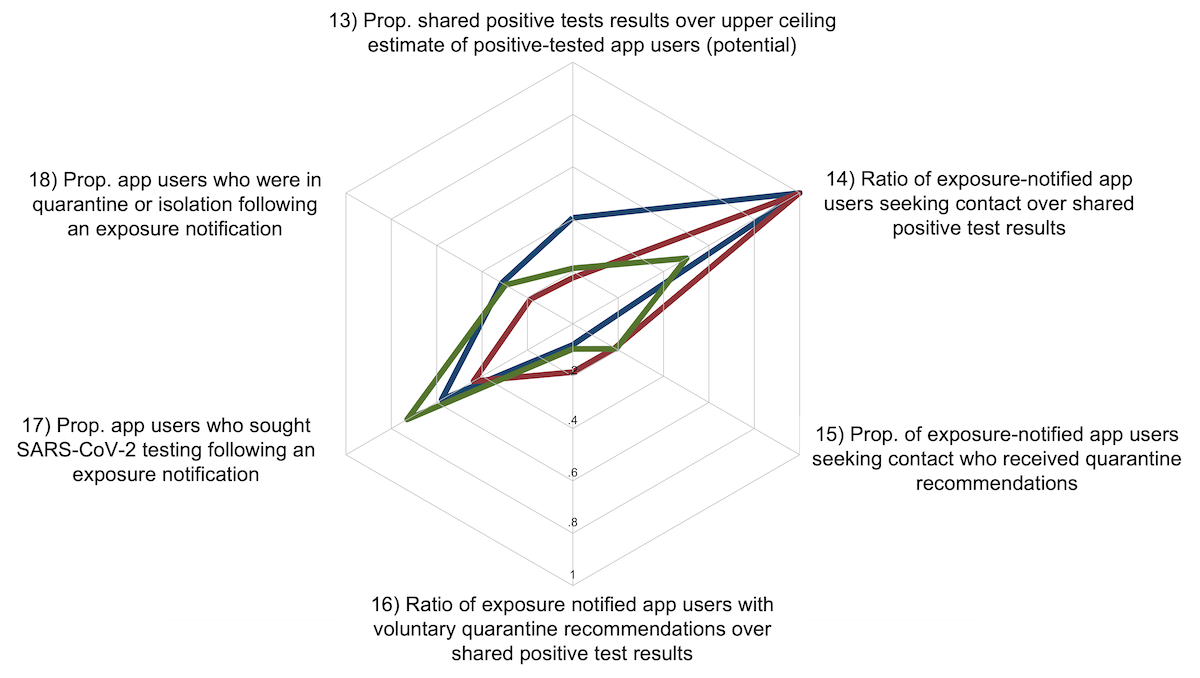
Indicators reflecting the probability of app user actions following exposure notifications or positive test results. The colored lines represent the Alpha (blue), Delta (red), and Omicron (green) variant phases. The plot ranges from 0 (center) to 1 (indicator 14 values were censored at 1, even though they were slightly higher; more information is available in Supplementary Table 2 in Multimedia Appendix 4) and illustrates the proportions and ratios of the relevant indicators. Indicator definitions and data sources are provided in Supplementary Table 1 in Multimedia Appendix 2. Prop.: proportion.

Indicators from the WHO/ECDC framework and selected complementary indicators from the COVID-19 Social Monitor data are illustrated in radar plots (Figures 2-4, data in Supplementary Tables 1 and 2 in Multimedia Appendix 2). Figure 2 illustrates indicators that relate to the performance of the exposure notification cascade (ie, completeness and speed of events). Starting with the top indicator and moving clockwise, indicator 1 shows the average weekly SARS-CoV-2 incidence from daily values (rescaled as percentage from the peak incidence). The maximum of daily case numbers was reached during the Omicron variant phase and the lowest daily case numbers during the Alpha variant phase. Indicator 2 shows that around 1 in 4 (1,779,546/7,280,501, 24.4%) Swiss individuals aged 16 years and older were active SwissCovid app users during the Alpha variant phase, while the percentage of SwissCovid app users decreased slightly during the Delta (1,624,946/7,280,501, 22.3%) and Omicron (1,568,104/7,280,501, 21.5%) variant phases.

Indicator 3 represents the number of authentication codes that were shared with the SwissCovid app as a fraction of the total number of individuals with a positive SARS-CoV-2 test. This percentage was 9.9% (20,273/204,741) during the Alpha variant phase and then declined to 3.9% (14,372/365,846) and 4.6% (72,324/1,581,506) during the Delta and Omicron variant phases, respectively. Indicator 4 reflects the ratio of authentication codes entered into the SwissCovid app over issued authentication codes. Here, we observed a nearly twice as large proportion of entered codes during the Alpha variant phase (20,273/31,658, 64%) compared with the Delta (14,372/44,455, 32.3%) and Omicron variant phases (72,324/269,700, 26.8%). Indicator 5 represents the timing of authentication code upload into the Swisscovid app from symptom onset or positive test date if the app user was asymptomatic at the time of testing. This indicator suggests that between 50% and 56% of all entered codes were uploaded within 48 hours after symptom onset, with lower percentages observed in the following 2 variant phases.

Lastly, indicator 6 represents the proportion of SwissCovid app users who completed the provided web form and called an infoline after receiving an exposure notification. Here, we observed that between 23% and 28% of exposure-notified app users contacted the infoline or completed the web form within 48 hours after the exposure date, which is provided in the exposure notification message.

#### Indicators Reflecting Test Positivity Following Exposure Notifications

Test positivity following receipt of an exposure notification is considered a proxy to assess the precision of exposure detection in notifying affected individuals. Figure 3 summarizes the key indicators in this context, although in a more refined scale, which ranges from 0 (0%) to 0.5 (50%). Indicator 7 illustrates test positivity in the general population, which was close to 10% in the first 2 variant phases (228,103/3,755,205, 6.1%, and 413,685/3,443,364, 12%, respectively) and increased to around 41.7% (1,784,951/4,285,549) in the Omicron variant phase. Indicators 7 and 8 are equivalent to indicators 2 and 3 in Figure 2. Indicator 8 represents the percentage of active app users, and indicator 9 represents the percentage of app users among individuals who tested positive, based on generated upload authentication codes. Indicator 10 illustrates the percentage of app users who received an exposure notification among all individuals with a positive test. This value was approximately 11% (7/65) in the Alpha variant phase and around 5% in the later 2 variant phases (2/44 and 11/228, respectively). Indicator 11 represents the percentage of app users who received an exposure notification among all app users who tested positive (calculated for indicator 10). Here, they were 19% (7/36) in the Alpha variant phase, 13% (2/15) in the Delta variant phase, and 8.3% (11/132) in the Omicron variant phase. Finally, indicator 12 illustrates test positivity among app users who received an exposure notification. This value was 19% (7/37) in the Alpha variant phase, 29% (2/7) in the Delta variant phase, and 41% (11/27) in the Omicron variant phase compared to 6.1% (228,103/3,755,205), 12% (413,685/3,443,364), and 41.7% (1,784,951/4,285,549) in the general population, respectively.

#### Indicators Reflecting User Actions Following Exposure Notifications

The third set of indicators illustrates the extent of mitigative actions taken by SwissCovid app users following receipt of exposure notifications. Figure 4 summarizes the key indicators in this context in a scale that ranges from scores 0 to 1. Indicator 13 illustrates the proportion of authorization codes entered into the SwissCovid app from individuals who tested positive by the upper ceiling estimate, which were 40.5% (20,273/50,044) for the Alpha variant, 17.6% (14,372/81,654) for the Delta variant, and 21.2% (72,324/340,631) for the Omicron variant.

Indicator 14 illustrates the ratio of users seeking contact through the infoline or completing the web form per shared positive test result. This value decreased over the course of the pandemic from 1.08 user contacts per code during the Alpha variant phase to 1.00 during the Delta variant phase and 0.50 during the Omicron variant phase. Indicator 15 illustrates the exposure risk assessment following contact with the infoline or via a web form, as well as a voluntary quarantine recommendation following receipt of exposure notifications. The proportion of quarantine recommendations per user contact was 7.4% (1622/21,976) during the Alpha variant phase and increased to 18.5% during the Delta and 19.1% during the Omicron variant phases (2652/14,313 and 6931/36,279, respectively).

Indicator 16 illustrates the standardized voluntary quarantine recommendations by the number of shared positive test results. Here, there were approximately 8 recommendations per 100 tests in the Alpha variant phase, 18 recommendations per 100 tests during the Delta variant phase, and 10 recommendations per 100 tests during the Omicron variant phase. Indicator 17 illustrates data from the COVID-19 Social Monitor and indicates that 58% (37/64), 44% (7/16), and 73% (27/37) app users sought testing or performed self-tests following an exposure notification during the Alpha, Delta and Omicron variant phases, respectively. Lastly, indicator 18 reveals that 31% (20/64, Alpha), 19% (3/16, Delta), and 30% (11/37, Omicron) of individuals who received exposure notifications also reported to have received mandatory quarantine orders by manual contact tracing or through a recommendation by a health care professional.

## Discussion

### Principal Findings

Our study presented various digital proximity-tracing app performance indicators for Switzerland. These were guided by and built upon the WHO/ECDC framework for the assessment of digital proximity-tracing apps' public health effectiveness in mitigating onward transmission of SARS-CoV-2. Our analysis extends the current knowledge in the field of digital proximity tracing by comparing various pandemic periods that were characterized by different SARS-CoV-2 variants of concern, as well as by changes in public perceptions of the pandemic and public health responses. Our study further contributes to effectiveness assessments on a methodological level by introducing further indicators of interest from panel survey data that assess mitigative strategies taken by individuals following receipt of exposure notifications. To the best of our knowledge, this is the first countrywide application of the WHO/ECDC performance assessment framework.

A first set of indicators explored the exposure notification cascade performance throughout the 3 variant phases. A substantially higher SARS-CoV-2 incidence was observed during the Omicron variant phase, while active SwissCovid app use steadily declined between the Alpha and the Omicron variant phases. Compared to the peak use of the SwissCovid app in early 2021 with nearly 2 million active app users, the numbers decreased by approximately 600,000 users in March 2022. Furthermore, the early months of 2022 were marked by not only the highest SARS-CoV-2 incidence in Switzerland but also the highest absolute numbers of shared positive test results throughout the whole pandemic. This led to capacity issues in Switzerland, since an insufficient number of SARS-CoV-2 tests were available to meet such high demands. Combined with the public perception of a lower disease severity of Omicron, these 2 factors have likely contributed to the lower percentage of shared test results in later pandemic phases. A further notable difference between the 3 variant phases was that a comparatively lower proportion of issued authentication codes were entered into the app with variants of concern that appeared later in the pandemic. This may have resulted from changes in authentication code–issuing practices throughout the pandemic phases (eg, by increasingly relying on automated delivery processes), as well as possibly by a decreased acceptance of the SwissCovid app [25].

The second set of indicators focused on general test positivity in Switzerland and the proportion of individuals who tested positive for SARS-CoV-2 upon receiving an exposure notification from the SwissCovid app. The indicators illustrated a close link between test positivity and the overall SARS-CoV-2 incidence in Switzerland throughout the different phases of the pandemic. Specifically, SARS-CoV-2 case numbers and test positivity were relatively low during the Alpha variant phase but increased during the Omicron variant phase. Our individual-level analyses suggested that test positivity after receiving an exposure notification was 2-3 times higher than in the general population in the Alpha and Delta variant phases and of similar magnitude (although at very high levels) during the Omicron variant phase. Even though this assessment is based on a relatively small sample size, the observed high test positivity is plausible in a wider context since the SwissCovid app operates on more conservative Bluetooth attenuation signal thresholds compared to the apps from other countries.

The third set of indicators suggests that the mitigative actions taken by app users following the receipt of an exposure notification from the SwissCovid app may have changed over the course of the pandemic. During the Omicron variant phase, fewer people contacted the infoline or completed web forms in comparison to the Alpha and Delta variant phases. This decrease in contact attempts also resulted in relatively fewer voluntary quarantine recommendations. In the Alpha and Omicron variant phases, the proportion of reports of entering into mandatory quarantine upon receiving an exposure notification was of similar magnitude. In contrast, a higher proportion of exposure-notified app users reported to have gotten tested throughout the earlier variant phases. This may have likely been due to shifts in public perceptions regarding the disease severity of SARS-CoV-2 over time. Furthermore, it could have been a response to changing public health strategies during the Omicron variant phase, such as removing mandatory quarantine for exposed contacts in Switzerland on February 17, 2022. As suggested by the high general test positivity of 40% during the Omicron variant phase, many symptomatic or exposed individuals also relied less on SARS-CoV-2 PCR testing but, rather, self-tested or just stayed at home. Since SARS-CoV-2–infected individuals who did not get tested at official testing centers did not receive upload authentication codes, they could consequently not share their test results with proximity contacts via the SwissCovid app.

The indicators also provide insights into the possible contribution of digital proximity-tracing apps, such as SwissCovid, in mitigating viral spread. For example, the ratio of shared positive test results over the upper ceiling estimate of positive tests among app users suggest that between 60% (Alpha variant phase) and 80% (Delta and Omicron variant phases) of estimated app users who tested positive did not or were unable to share their test results. The reasons for this may include that a lower number of issued authentication codes were entered into the SwissCovid app or that there were delays in issuing authentication codes. The latter can negatively affect the potential for digital proximity tracing if exposed contacts are informed faster through other means (eg, if the number of potential contacts is small or well known and can be reached efficiently by manual contact tracing). Nevertheless, the SwissCovid app has been shown to have advantages in timeliness and efficacy in users taking mitigative actions over manual contact tracing in recent studies. For example, 1 study revealed that app users who received an exposure notification from the SwissCovid app entered quarantine, on average, 1 day earlier than contacts who did not receive an exposure notification [16]. A simulation conducted in another study similarly found that 5% of people in manual contact tracing–mandated quarantine entered isolation after receiving a voluntary quarantine recommendation from an exposure notification [8]. The usefulness of both strategies to enable effective contact tracing can be, however, diminished by incomplete user actions. This was not observed in our study, where we found that relatively few app users who received exposure notifications ignored the exposure warning. Most of these app users undertook at least 1 recommended mitigative step in response to the notification, such as calling the infoline or completing the web form, which is in line with other studies from Switzerland [26,27].

Furthermore, relevant actions for transmission prevention were also quite frequently reported, as almost 3 out of 4 exposure-notified SwissCovid app users reported getting tested or having entered quarantine during the Omicron variant phase. These estimates fall in line with other studies using the same [28] or different Swiss survey databases [29]. However, they could be prone to reporting biases, such as social desirability bias, characterized as the tendency of survey respondents to answer questions in a manner that will be viewed favorably by others. In addition, an apparent lack of response to exposure notifications may also be due to the timing of the notification or the exposed app users’ varying individual assessments of possible exposure settings and severity of transmission risks. For example, detailed reports from a Swiss study demonstrated that delayed notification, within-household exposures, or the application of preventive measures at time of exposure may be reasons for not responding to exposure notifications (Zurich Coronavirus Cohort [ZSAC]) [8].

Overall, our study contributes to the accumulating evidence of the possible contribution of digital proximity-tracing apps toward pandemic mitigation through quantitative evidence within an established public health indicator framework. However, our study also indicates various shortcomings of digital proximity-tracing apps that interfere with their ability to function at their full potential. In the case of the SwissCovid app, the flow of information along the exposure notification cascades was limited by various bottlenecks, such as delayed code delivery for test result sharing, complex user interfaces, or misaligned incentives for subsequent mitigative actions. This was observed with the SwissCovid app use visibly decreasing over time despite increasing prevalence with the more recent SARS-CoV-2 variants. The bottlenecks that may have contributed to decreased use of the SwissCovid app were recently illustrated by a study where case-contact pairs fulfilled all necessary conditions to enable exposure notifications (ie, use of the SwissCovid app, sharing of test results), but only 6 of 10 exposed contacts ended up receiving exposure notifications [26]. To enable future large-scale implementations of digital proximity-tracing apps, further testing of such apps under higher-capacity requirements, as well as co-design processes in app development, may be beneficial.

### Limitations

Our study bears some limitations. The data and assessment methods used in this analysis cannot provide evidence for causality between digital proximity-tracing app use and transmission prevention. Due to a lack of clinical outcomes data, our findings are also not suited to extrapolate the population-level impact of digital proximity-tracing apps, such as avoided hospitalizations or deaths due to a lack of clinical outcome data. Moreover, despite drawing on an extensive database that includes almost 2700 individuals and 23,500 assessments, the number of recorded events of interest (ie, exposure notifications, positive SARS-CoV-2 tests, quarantine mandates) was still relatively low. This is a common issue of population-based surveys, where the probability of occurrence at any time point remains small and thus rather represents a general methodological challenge in such research. Finally, survey-driven studies may be prone to different reporting biases, including over- or underreporting of mitigative behaviors, such as noncompliance with rules and social norms. However, this was to a degree mitigated by the longitudinal nature of our data collection and repeated surveying of SwissCovid app use and outcomes, which allowed for various quality checks and did not reveal indications for systematic reporting biases.

### Conclusion

Our study provides a comprehensive countrywide assessment of key indicators for the SwissCovid digital proximity-tracing app based on the WHO/ECDC framework and highlights the importance of considering the overall pandemic context in the assessment of the performance and effectiveness of such apps. For example, test positivity upon receipt of an exposure notification from the SwissCovid app was at least as high as (Omicron variant phase) or higher than (Alpha and Delta variant phases) general test positivity, with a high percentage of app users taking mitigative actions upon receiving an exposure notification. Furthermore, more than 200,000 individuals shared positive test results with the app over the course of the pandemic. Nevertheless, our indicator assessment also suggests room for improvement, including improving the speed and completeness of the exposure notification cascade or establishing stronger incentives for app use and test result sharing. Future implementations of digital proximity-tracing apps should place more emphasis on the social, psychological, and organizational aspects of the exposure notification cascade to improve their effectiveness in mitigating pandemic spread. In the context of constantly evolving requirements across different pandemic waves, the implementation of digital proximity-tracing apps should be regularly reviewed and revised.

## Appendix

Multimedia Appendix 1 Exposure notification cascade in Switzerland and related indicators.

Multimedia Appendix 2 Description and assessments of indicators.

Multimedia Appendix 3 Description of data sources, assessments, and pandemic context.

Multimedia Appendix 4 Study population and participant characteristics.

Multimedia Appendix 5 Description of Venn diagram and subpopulations.

Multimedia Appendix 6 Description and assessments of indicators.

## Abbreviations

DP-3T: Decentralized Privacy-Preserving Proximity Tracing
ECDC: European Center for Prevention and Disease Control
PCR: polymerase chain reaction
WHO: World Health Organization

## Data Availability

Data used from the COVID-19 Social Monitor study and from Medgate Aktiengesellschaft are available upon request from the corresponding author. All other data sources are publicly available.
