# Supplementary material for: Performance of the Swiss Digital Contact-Tracing App Over Various SARS-CoV-2 Pandemic Waves: Repeated Cross-sectional Analyses
Source: JMIR Public Health Surveill. 2022 Nov 11;8(11):e41004. doi: 10.2196/41004 (PMC9700234; doi:10.2196/41004)

**Multimedia Appendix 1:** Exposure notification cascade in Switzerland and related indicators

**Supplementary Figure 1a**: Exposure notification cascade as implemented in Switzerland


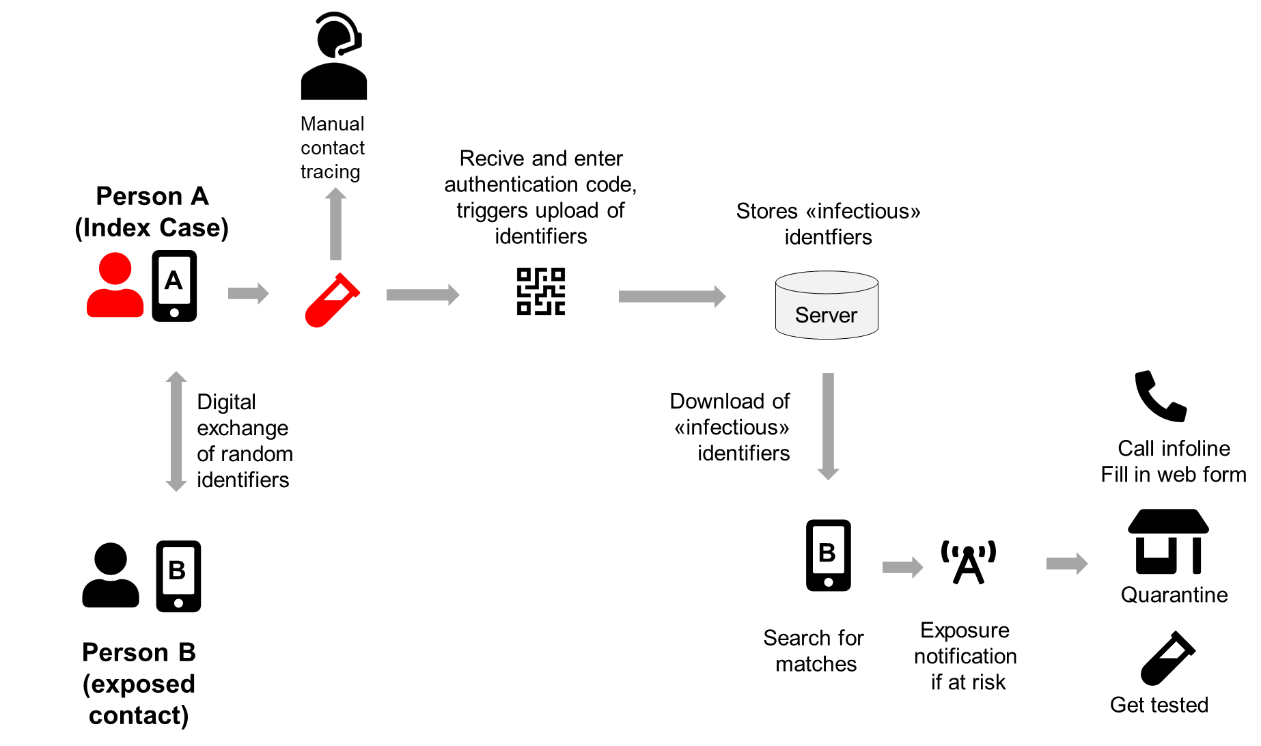


The exposure notification cascade illustrates the information flow, points of contact of app users, and recommended actions upon exposure notification. Person A has had a proximity encounter with person B. Both were using the digital proximity tracing app at the time of contact, and pseudonymized identification numbers were exchanged by the apps. Person A later tests positive for SARS-CoV-2 and receives an upload authentication code from manual contact tracing or via a call from a separate infoline. This person’s pseudonymized identification numbers are uploaded onto a central server upon sharing the positive test result (i.e., entering of the authentication code). These identification numbers are downloaded by all other app users, including person B, on a regular basis. Person B’s app searchers for matches of downloaded “infectious” identifiers with the identification numbers stored from its encounter history (up to the last 14 days). If a match is found, person B receives an exposure notification alert, which includes a link to a self-assessment online form and the phone number to an infoline. Following the exposure risk assessment via the web form or through the infoline, the exposed person may be recommended to remain in voluntary quarantine. Persons receiving an exposure notification are also entitled to a free SARS-CoV-2 test.

**Supplementary Figure 1b**: Exposure notification cascade in Switzerland adapted for ECDC/WHO framework “A” indicators (adoption of the SwissCovid app and exposure notification trigger)


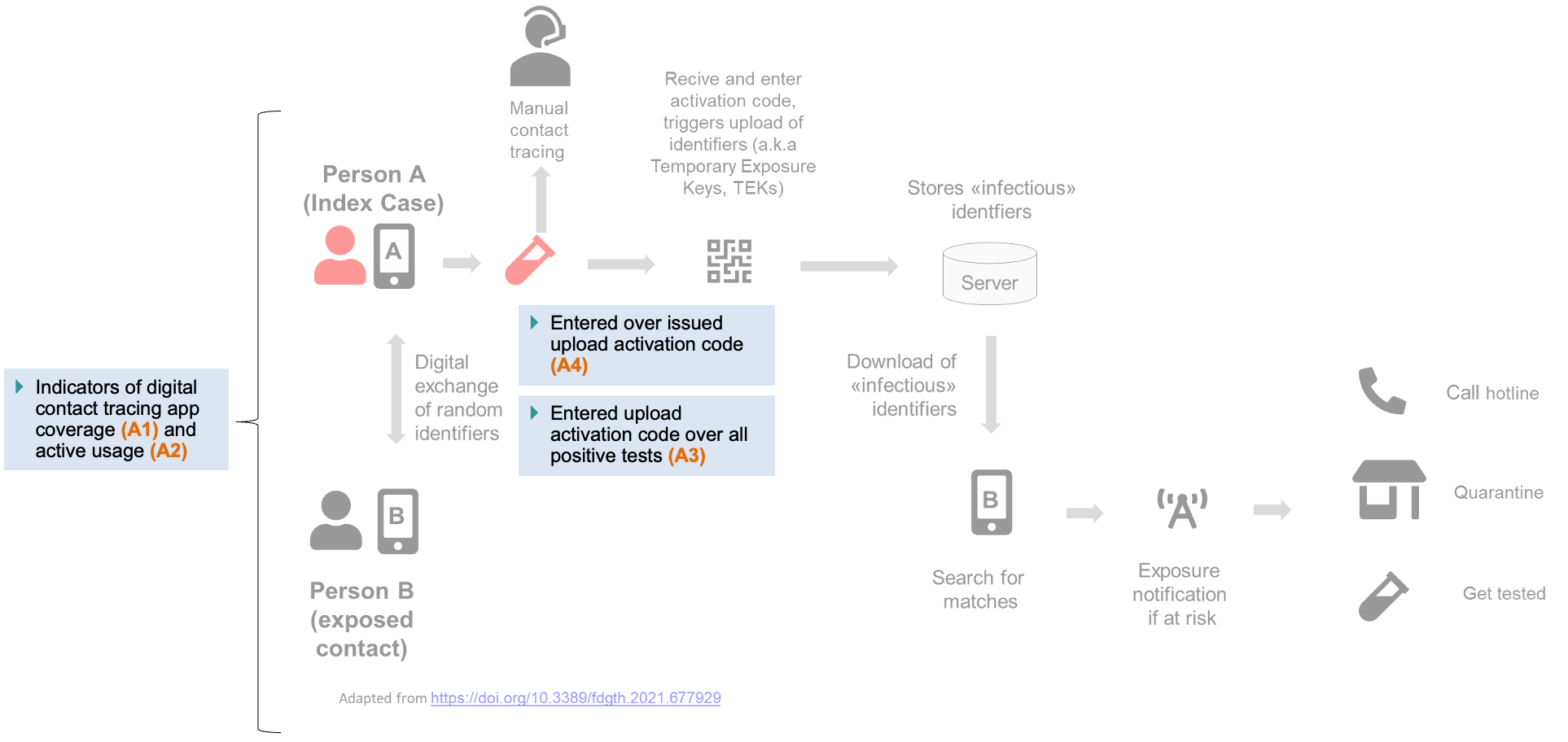


**Supplementary Figure 1c**: Exposure notification cascade in Switzerland adapted for ECDC/WHO framework “B” indicators (exposure notification and testing for SARS-CoV-2)


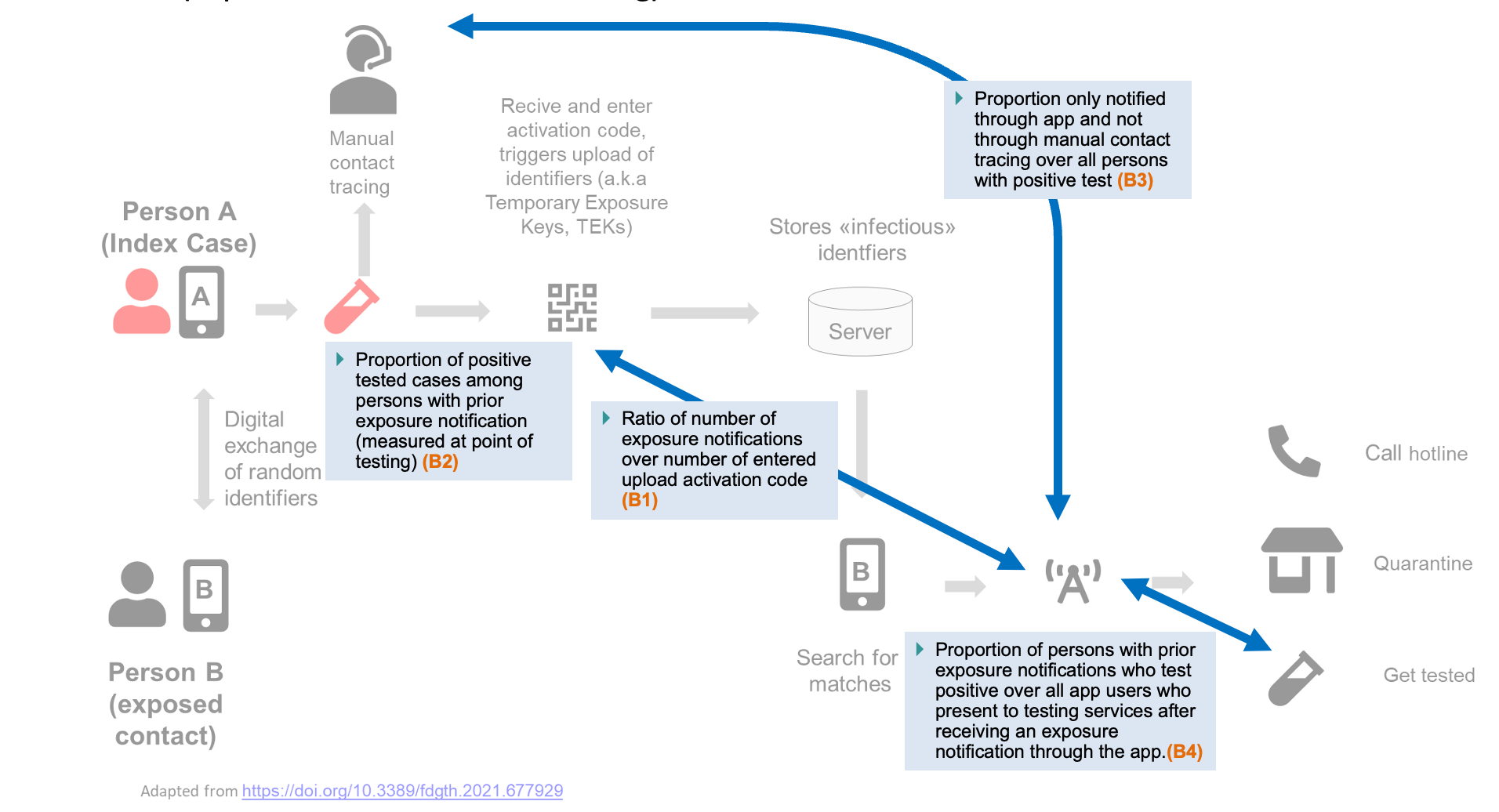


**Supplementary Figure 1d**: Exposure notification cascade in Switzerland adapted for ECDC/WHO framework “C” indicators (timeliness and comparative speed advantage of exposure notifications)


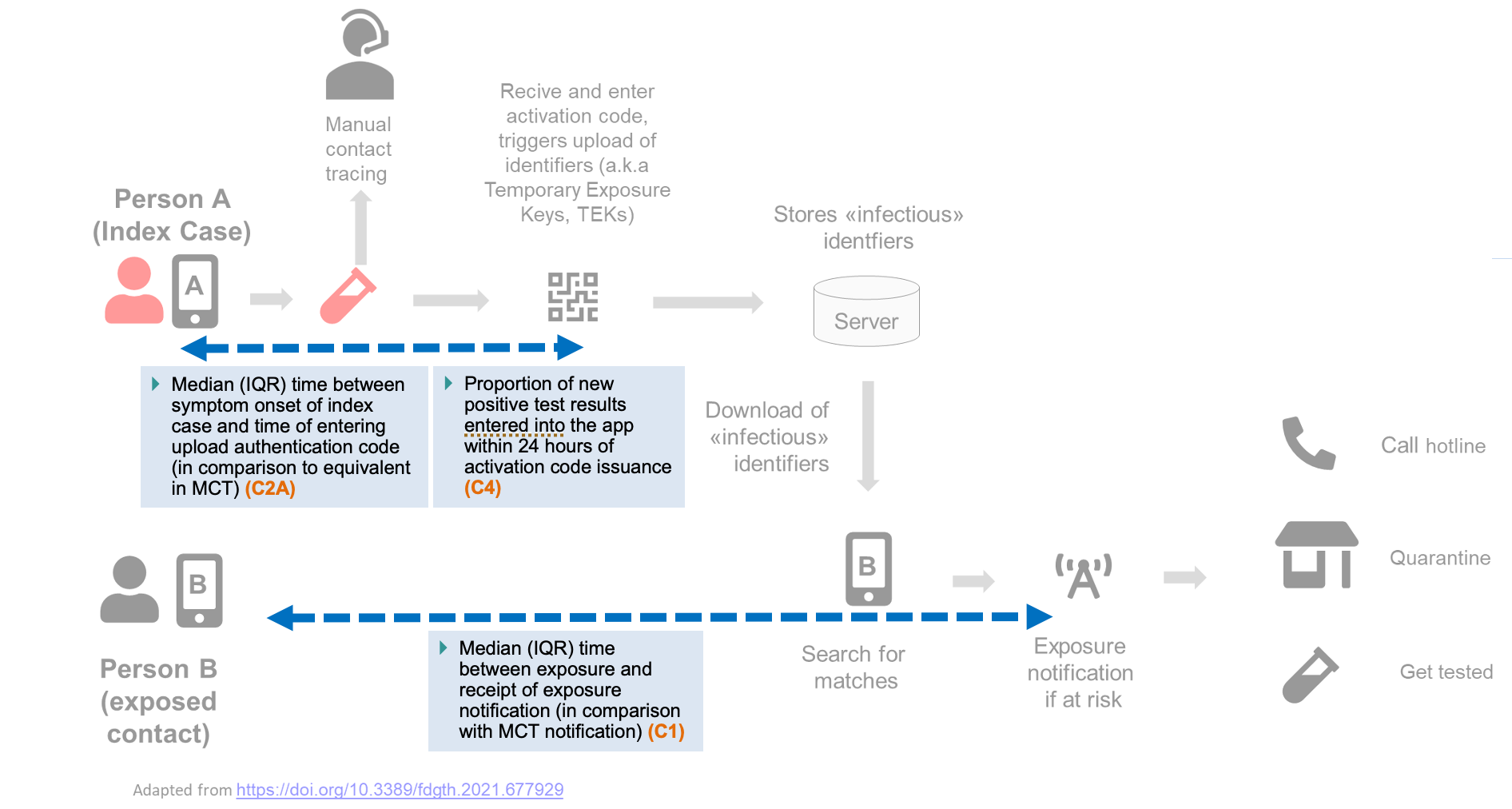


**Supplementary Figure 1e**: Exposure notification cascade in Switzerland adapted for ECDC/WHO framework “D” indicators (factors associated with SwissCovid app adoption)


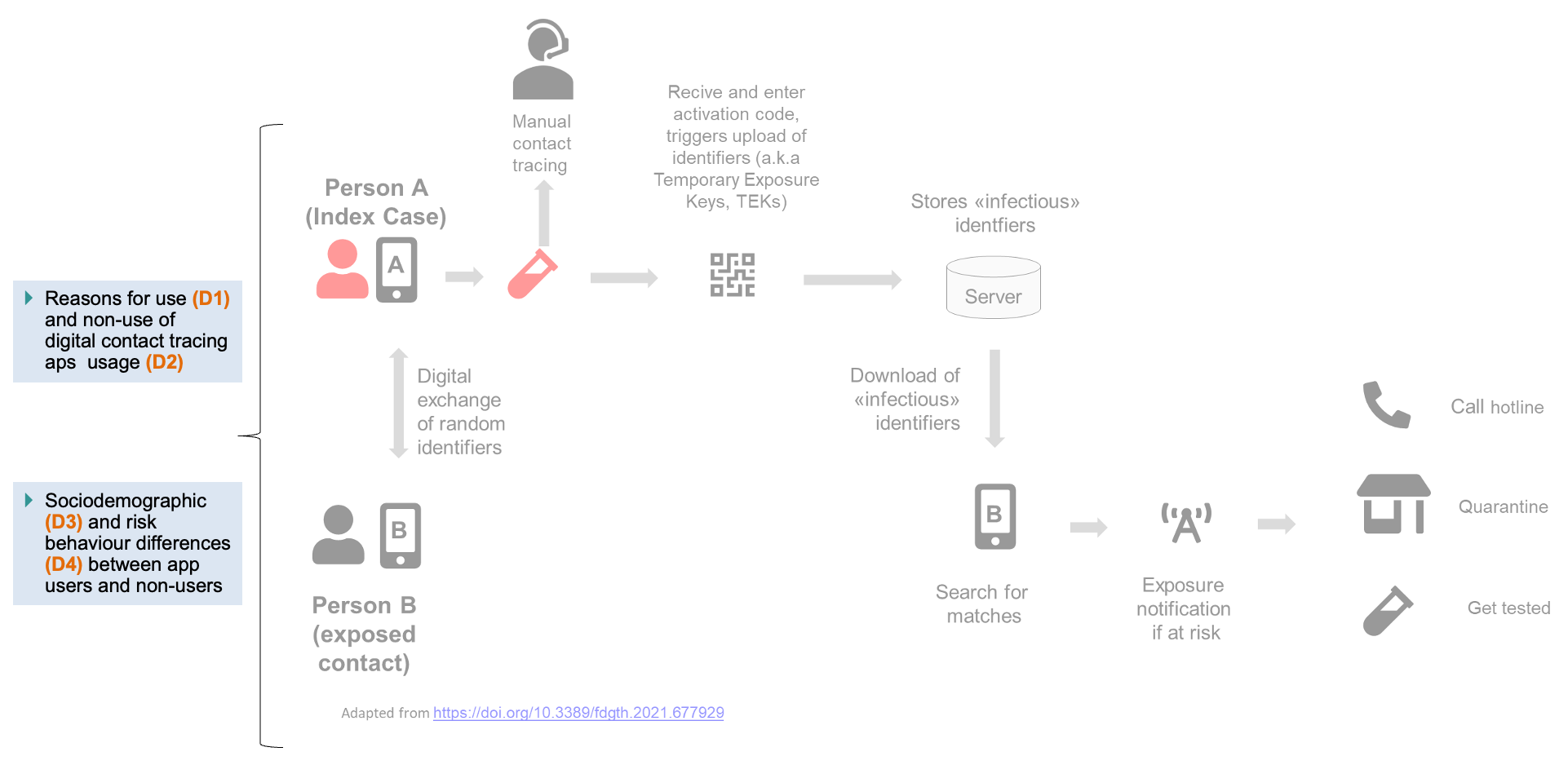

Supplement: Multimedia Appendix 1 [file publichealth_v8i11e41004_app1.docx]
