# Supplementary material for: Performance of the Swiss Digital Contact-Tracing App Over Various SARS-CoV-2 Pandemic Waves: Repeated Cross-sectional Analyses
Source: JMIR Public Health Surveill. 2022 Nov 11;8(11):e41004. doi: 10.2196/41004 (PMC9700234; doi:10.2196/41004)
Supplement: Multimedia Appendix 2 [file publichealth_v8i11e41004_app2.docx]

**Multimedia Appendix 2**: Description and assessments of indicators

**Supplementary Table 1**: Description of indicators and relevant sources

| **Topic** | **Indicator number** | **Description** | **Definition** | **Interpretation** | **WHO/**  **ECDC indicator** | **Alpha** | **Delta** | **Omicron** | **Data** |
| --- | --- | --- | --- | --- | --- | --- | --- | --- | --- |
| Cascade Performance | 1 | Incidence of SARS-CoV-2 in Switzerland | Number of positive SARS-CoV-2 tests, averaged over calendar week (proportion, relative to peak incidence) | Contextual parameter. A higher incidence means a greater likelihood that capacity limits of manual contact tracing and public health services are reached. | n.a. | 0.13 | 0.23 | 1.00  (ref.) | FOPH |
| (Figure 2) | 2 | Percentage of app users in Swiss population | Number of active app users as a fraction of the number of Swiss residents aged 16 years and older | A higher percentage of app users will lead to more shared test results. The probability for an exposure notification is proportional to the square power of this percentage.[1] | A2 | 0.24 | 0.22 | 0.22 | FOS |
|  | 3 | Percentage of shared positive test results over all positive tests | Number of entered authentication codes over all positive test results | The higher this proportion the more exposure contacts can be notified. This indicator is dependent on app coverage. | n.a. | 0.10 | 0.04 | 0.05 | FOS |
|  | 4 | Percentage of shared positive test results | Number of entered authentication codes over number of issued authentication codes | If the number of entered over issued authentication codes is low, this could be suggestive of barriers in code issuance or disincentives for code entering. The indicator also depends on code delivery practices. For example, some countries deliver authentication codes for all positive tests, regardless of app usage. | A4 | 0.64 | 0.32 | 0.27 | Infoline FOS |
|  | 5 | Percentage of positive tests shared within 48 hours after symptom onset or detection (if pre- or asymptomatic) | Number of days between symptom onset and authentication code upload | Faster sharing of positive test results decreases the likelihood for further onward transmission by exposed contacts. | C4, modified | 0.56 | 0.50 | 0.52 | FOS |
|  | 6 | Percentage of exposure notifications received within 48 hours after exposure | Number of days between exposure date (as provided in exposure notification message) and infoline contact or web form completion | Faster receipt of an exposure notification decreases the likelihood for further onward transmission by exposed contacts. This parameter must be interpreted with caution because many contacts could not provide any information on the estimated time frame between exposition and call. This parameter also only include persons who decided to call the infoline. | n.a. | 0.28 | 0.23 | 0.26 | Infoline |
| Test  Positivity | 7 | Test positivity in general population | Number of positive SARS-CoV-2 tests over all tests performed | Percentage of positive tests. The WHO recommends that test positivity should not exceed 5%.[2] | n.a. | 0.06 | 0.12 | 0.42 | FOPH |
| (Figure3) | 8 (same as 2) | Percentage of app users in Swiss population | Number of active apps as a fraction of the number of Swiss residents aged 16 years and older |  | A2 | 0.24 | 0.22 | 0.22 | FOS |
|  | 9 (same as 3) | Percentage of shared positive test results over all positive tests | Number of entered authentication codes over all positive test results |  | n.a. | 0.10 | 0.04 | 0.05 | FOS |
|  | 10 | Percentage of persons with exposure notifications among all positive tested persons | Venn notation (including app user only): (B+C)/(A+B+C+D) | This indicator is a proxy for the proportion of positive tests that may potentially have been detected due to exposure notifications. It must be interpreted with caution, however, because people may have sought testing reasons other than the exposure notification. | B3 | 0.11 | 0.05 | 0.05 | Soc Mon |
|  | 11 | Percentage of persons with exposure notifications among all positive tested app users | Venn notation (including all persons): (B+C)/[(A+B+C+D)] | Could be indicative for what percentage of positive tested app users may have responded to exposure notifications and sought testing. | B2 | 0.19 | 0.13 | 0.08 | Soc Mon |
|  | 12 | Test positivity among app users with exposure notification | Venn notation: (B+C)/[(B+C+E1+F1)] | If the test positivity in exposure- notified persons is higher than in the general population, this may be informative about the specificity of exposure risk detection by digital proximity tracing in the sense that relevant risks are detected. | B4 | 0.19 | 0.29 | 0.41 | Soc Mon |
| User Actions (Figure 4) | 13 | Percentage of shared positive tests results over upper ceiling estimate of positive tested app users (potential) | Percentage of active app users, multiplied by the total number of positive SARS-CoV-2 tests | This indicator approximate the number of shared tests among positive tested app users. The number of positive tested app users is interpolated by multiplying the number of positive tested persons by the observed percentage of active app users in the Swiss population. | n.a. | 0.41 | 0.18 | 0.21 | FOS |
|  | 14 | Ratio of the registered infoline calls or completed web forms over shared test results | Number of infoline calls or completed web forms over entered authentication codes | This indicator measures the fraction of exposure notified persons who take the notification seriously and undertake actions. The parameter does not capture persons who seek testing but do not call the infoline, however.  This parameter is highly context-dependent and is influenced, for example, by public health recommendations, the fraction of persons calling the infoline, and specificity of exposure notifications. | n.a. | 1.08 | 1.00 | 0.50 | FOS |
|  | 15 | Percentage of voluntary quarantine recommendations among all callers/web form completers | Number of quarantine recommendations over number of callers or web completers | A higher percentage of quarantined persons following exposure notification decreases the likelihood for onward transmission. This parameter is highly context-dependent and is influenced, for example, by public health recommendations, the fraction of persons calling the infoline, and specificity of exposure notifications. | n.a. | 0.07 | 0.19 | 0.19 | Infoline FOS |
|  | 16 | Ratio of exposure-notified app users with voluntary quarantine recommendations over shared SARS-CoV-2 test results | Number of quarantine recommendations over number of entered authentication codes | A higher percentage of quarantined persons following exposure notification decreases the likelihood for onward transmission. This parameter mainly reflects the behavior of exposure notified app users is highly context-dependent and is influenced, for example, by public health recommendations and specificity of exposure notifications. | n.a. | 0.08 | 0.18 | 0.10 | Infoline FOS |
|  | 17 | Percentage of app users who sought testing following an exposure notification | Venn notation: [(B+C+E1+F1)]/[(B+C+E1+E2+F1+ F2)] | A higher percentage of persons seeking testing after an exposure notification decreases the likelihood for onward transmission. This parameter is highly context-dependent and is influenced, for example, by public health recommendations. | n.a. | 0.58 | 0.44 | 0.73 | Soc Mon |
|  | 18 | Percentage of app users who were in quarantine or isolation following an exposure notification | Venn notation: [(C+F1)]/[(B+C+E1+E2+F1+ F2)] | A higher percentage of quarantined persons following exposure notification decreases the likelihood for onward transmission. This parameter is highly context-dependent and is influenced, for example, by public health recommendations and specificity of exposure notifications. | n.a. | 0.31 | 0.19 | 0.30 | Soc Mon |

**Abbreviations** : FOS : Federal Office of Statistics, FOPH: Federal Office of Public Health, Soc Mon: COVID-19 Social Monitor, WHO/ECDC World Health Organization and European Center for Disease Control, [] : SwissCovid app user subpopulations.

**Supplementary Table 2**: Assessment of indicators

| **Indicator number** | **Numerator (Alpha)** | **Denominator (Alpha)** | **Alpha** | **Numerator (Delta)** | **Denominator (Delta)** | **Delta** | **Numerator (Omicron)** | **Denominator (Omicron)** | **Omicron** |  |
| --- | --- | --- | --- | --- | --- | --- | --- | --- | --- | --- |
|  |  |  |  |  |  |  |  |  |  |  |
| 1 | 204741 | 1581506 | 13% [95% CI: 13% - 13%] | 365846 | 1581506 | 23% [95% CI: 23% - 23%] | 1581506 | 1581506 | 100% [95% CI: 100%-100%] |  |
|  |  |  |  |  |  |  |  |  |  |  |
| 2 | 1779546 | 7280501 | 24% [95% CI: 24% - 25%] | 1624946 | 7280501 | 22% [95% CI: 22% - 22%] | 1568104 | 7280501 | 22% [95% CI: 22% - 22%] |  |
| 3 | 20273 | 204741 | 9.9% [95% CI: 9.9% - 10%] | 14372 | 365846 | 3.9% [95% CI: 3.9% - 4.0%] | 72324 | 1581506 | 4.6% [95% CI: 4.5% - 4.6%] |  |
| 4 | 20273 | 31658 | 64% [95% CI: 64% - 65%] | 14372 | 44455 | 32% [95% CI: 32% - 33%] | 72324 | 269700 | 27% [95% CI: 27% - 27%] |  |
|  |  |  |  |  |  |  |  |  |  |  |
| 5 |  |  | 56.4% |  |  | 49.7% |  |  | 52.0% |  |
|  |  |  |  |  |  |  |  |  |  |  |
| 6 |  |  | 28.2% |  |  | 23.1% |  |  | 25.6% |  |
| 7 | 228103 | 3755205 | 6.1% [95% CI: 6.1% - 6.1%] | 413685 | 3443364 | 12% [95% CI: 12% - 12%] | 1784951 | 4285549 | 42% [95% CI: 42%-42%] |  |
|  |  |  |  |  |  |  |  |  |  |  |
| 8 (same as 2) | 1779546 | 7280501 | 24% [95% CI: 24% - 25%] | 1624946 | 7280501 | 22% [95% CI: 22% - 22%] | 1568104 | 7280501 | 22% [95% CI: 22%-22%] |  |
|  |  |  |  |  |  |  |  |  |  |  |
| 9 (same as 3) | 20273 | 204741 | 9.9% [95% CI: 9.8% - 10%] | 14372 | 365846 | 3.9% [95% CI: 3.9% - 4.0%] | 72324 | 1581506 | 4.6% [95% CI: 4.5% - 4.6%] |  |
|  |  |  |  |  |  |  |  |  |  |  |
| 10 | 7 | 65 | 11% [95% CI: 4.4% - 20.9%] | 2 | 44 | 4.5% [95% CI: 0.6% - 16%] | 11 | 228 | 4.8% [95% CI: 2.4%-8.5%] |  |
| 11 | 7 | 36 | 19% [95% CI: 8.2% - 36%] | 2 | 15 | 13% [95% CI: 1.7% - 41%] | 11 | 132 | 8.3% [95% CI: 4.2% - 14%] |  |
| 12 | 7 | 37 | 19% [95% CI: 8.0% - 35%] | 2 | 7 | 29% [95% CI: 3.7% - 71%] | 11 | 27 | 41% [95% CI: 22% - 61%] |  |
| 13 | 20273 | 50044 | 41% [95% CI: 40% - 41%] | 14372 | 81654 | 18% [95% CI: 17% - 18%] | 72324 | 340631 | 21% [95% CI: 21%-21%] |  |
|  |  |  |  |  |  |  |  |  |  |  |
| 14 | 21976 | 20273 | 1.08 | 14313 | 14372 | 1.00 | 36279 | 72324 | 0.50 |  |
| 15 | 1622 | 21976 | 7.4% [95% CI: 7.0% - 7.7%] | 2652 | 14313 | 19% [95% CI: 18% - 19%] | 6931 | 36279 | 19% [95% CI: 19% - 20%] |  |
|  |  |  |  |  |  |  |  |  |  |  |
| 16 | 1621 | 20273 | 8.0% [95% CI: 7.6% - 8.4%] | 2650 | 14372 | 18% [95% CI: 18% - 19%] | 6931 | 72324 | 9.6% [95% CI: 9.4% - 9.8%] |  |
|  |  |  |  |  |  |  |  |  |  |  |
| 17 | 37 | 64 | 58% [95% CI: 45% - 70%] | 7 | 16 | 44% [95% CI: 20% - 70%] | 27 | 37 | 73% [95% CI: 56%-86%] |  |
| 18 | 20 | 64 | 31% [95% CI: 20% - 44%] | 3 | 16 | 19% [95% CI: 4.0% - 46%] | 11 | 37 | 30% [95% CI: 16%-47%] |  |
