# Supplementary material for: Performance of the Swiss Digital Contact-Tracing App Over Various SARS-CoV-2 Pandemic Waves: Repeated Cross-sectional Analyses
Source: JMIR Public Health Surveill. 2022 Nov 11;8(11):e41004. doi: 10.2196/41004 (PMC9700234; doi:10.2196/41004)
Supplement: Multimedia Appendix 3 [file publichealth_v8i11e41004_app3.docx]

**Multimedia Appendix 3**: Description of data sources, assessments, and pandemic context

The COVID-19 Social Monitor Study

The COVID-19 Social Monitor study collects indicators on the well-being, physical and mental health, health behavior, and employment situation of the Swiss population in the context of the COVID-19 pandemic. Information collected by the COVID-19 Social Monitor study include the use of the SwissCovid app and receipt of exposure notifications[1], mitigative actions taken by respondents (including entering quarantine), SARS-CoV-2 testing, and COVID-19 vaccination uptake. These questions were developed and reviewed by experts.[2]

The Cantonal Ethics Commission of Zurich concluded that the current study does not fall within the scope of the Human Research Act (BASEC-Nr. Req-2020-00323). As per this decision, explicit informed consent was not needed from participants for this particular study. However, participation in the COVID-19 Social Monitor study was voluntary and participants could withdraw from the study at all times.

The COVID-19 Social Monitor study cohort comprises of Swiss residents aged 18 to 79 years who were able and willing to complete online surveys. Participants for this cohort were randomly sampled from an existing web-based panel from a Swiss survey company.[3] Sampling for this cohort was stratified by age, gender, and language region, and is representative of the Swiss resident population in this regard (based on the 2018 census). The study was launched in March 2020, with 22 survey waves, as of May 2022, which were offered on a weekly basis in the early phase of the pandemic and then every 4 to 6 weeks. The initial sample that recruited participants in March 2020 comprised 2’026 participants and was replenished with an additional sample of 1,355 participants recruited in December 2020 from the same market research panel to counteract panel attrition.

We assessed data from the COVID-19 Social Monitor study cohort collected in December 2020 (baseline) until March 2022 (follow-up period). The baseline cohort from December 2020 is from study wave 12, which also includes the first study visit for the replenished cohort, and the follow-up period encompasses 10 further study waves. For study inclusion, at least one follow-up assessment was required from each participant included at the baseline (**Supplementary Figure 2**). For study exclusion, participants who reported having received an exposure notification at or before the baseline were not considered in our analysis. Furthermore, our analysis only considers the first exposure notification event indicated by the study participants. Mitigative actions and other relevant events following an exposure notification (i.e., SARS-CoV-2 testing, calling the infoline, entering quarantine, receipt of positive test results) were only considered in our analysis if they were mentioned in the same or in the immediately following study wave, since test results and quarantine orders may be received with some delay.

Using a Venn diagram-based approach for indicator calculation

Indicators reflecting user actions upon exposure notification (including test positivity after exposure notification, test positivity among app users) were derived from individual-level data collected by the Social Monitor study. These data were aggregated and analyzed using a Venn diagram –based approach[4,5] (**Supplementary Figure 3**) with the following four attributes: having tested positive for SARS CoV-2, having been tested for SARS-CoV-2, having been in isolation or quarantine ordered by a physician or manual contact tracing, and having received an exposure notification. All indicator calculations were stratified by three time periods reflecting the dominance of three different viral variants of concern[6] and aligned with Social Monitor survey assessments: Alpha variant (January 25, 2021 to June 17, 2021, survey waves 13 to 17), Delta variant (August 30, 2021 to December 16, 2021; survey waves 18 to 20), and Omicron BA.1 variant (January 24, 2022 – March 19, 2022; survey waves 21 to 22). The data were utilized to estimate the percentage of persons with exposure notifications among all positive tested persons (**Supplementary Table 3**, indicator 10), the percentage of persons with exposure notifications among all positive tested app users (indicator 11), and the test positivity among app users with exposure notification (indicator 12).

SARS-CoV-2 incidence during the study period

Taking the mean of daily SARS-CoV-2 case counts over the three periods of interest, there were an average of 1442 cases daily during the Alpha period, 3934 cases during the Delta period, and 25508 cases during the Omicron period. When standardized by the Swiss population (8670300 inhabitants), these amount to 16.6 daily cases per 100’000 persons, 45.4 daily cases per 100’000 persons, and 294.2 new daily cases per 100’000 persons.[6]

**References**

1. Wyl V von, Höglinger M, Sieber C, Kaufmann M, Moser A, Serra-Burriel M, Ballouz T, Menges D, Frei A, Puhan MA. Drivers of Acceptance of COVID-19 Proximity Tracing Apps in Switzerland: Panel Survey Analysis. JMIR Public Health Surveill 2021 Jan 6;7(1):e25701. [doi: 10.2196/25701]

2. Speierer A, Chocano-Bedoya PO, Anker D, Schmid A, Keidel D, Vermes T, Imboden M, Levati S, Franscella G, Corna L, Amati R, Harju E, Luedi C, Michel G, Veys-Takeuchi C, Zuppinger C, Nusslé SG, D’Acremont V, Tall I, Salberg É, Baysson H, Lorthe E, Pennacchio F, Frei A, Kaufmann M, Geigges M, West EA, Schwab N, Cullati S, Chiolero A, Kahlert C, Stringhini S, Vollrath F, Probst-Hensch N, Rodondi N, Puhan MA, von Wyl V. The Corona Immunitas Digital Follow-Up eCohort to Monitor Impacts of the SARS-CoV-2 Pandemic in Switzerland: Study Protocol and First Results. Int J Public Health [Internet] Frontiers; 2022 [cited 2022 Jun 9];0. [doi: 10.3389/ijph.2022.1604506]

3. The Swiss Leader for Social & Market Research [Internet]. LINK. [cited 2022 Jun 9]. Available from: https://www.link.ch/en/

4. Lueks W, Benzler J, Bogdanov D, Kirchner G, Lucas R, Oliveira R, Preneel B, Salathé M, Troncoso C, von Wyl V. Toward a Common Performance and Effectiveness Terminology for Digital Proximity Tracing Applications. Front Digit Health [Internet] 2021 [cited 2022 Jun 13];3. Available from: https://www.frontiersin.org/article/10.3389/fdgth.2021.677929

5. Daniore P, Nittas V, Moser A, Höglinger M, Wyl V von. Using Venn Diagrams to Evaluate Digital Contact Tracing: Panel Survey Analysis. JMIR Public Health Surveill 2021 Dec 6;7(12):e30004. [doi: 10.2196/30004]

6. COVID-⁠19 Switzerland | Coronavirus | Dashboard [Internet]. [cited 2021 Apr 1]. Available from: https://www.covid19.admin.ch/en/overview
