# Supplementary material for: Performance of the Swiss Digital Contact-Tracing App Over Various SARS-CoV-2 Pandemic Waves: Repeated Cross-sectional Analyses
Source: JMIR Public Health Surveill. 2022 Nov 11;8(11):e41004. doi: 10.2196/41004 (PMC9700234; doi:10.2196/41004)
Supplement: Multimedia Appendix 4 [file publichealth_v8i11e41004_app4.docx]

**Multimedia Appendix 4**: Study population and participant characteristics

**Supplementary Figure 2**: Flowchart of data selection for the calculation of Venn diagram-based indicators based on individual-level information collected by the COVID-19 Social Monitor study.


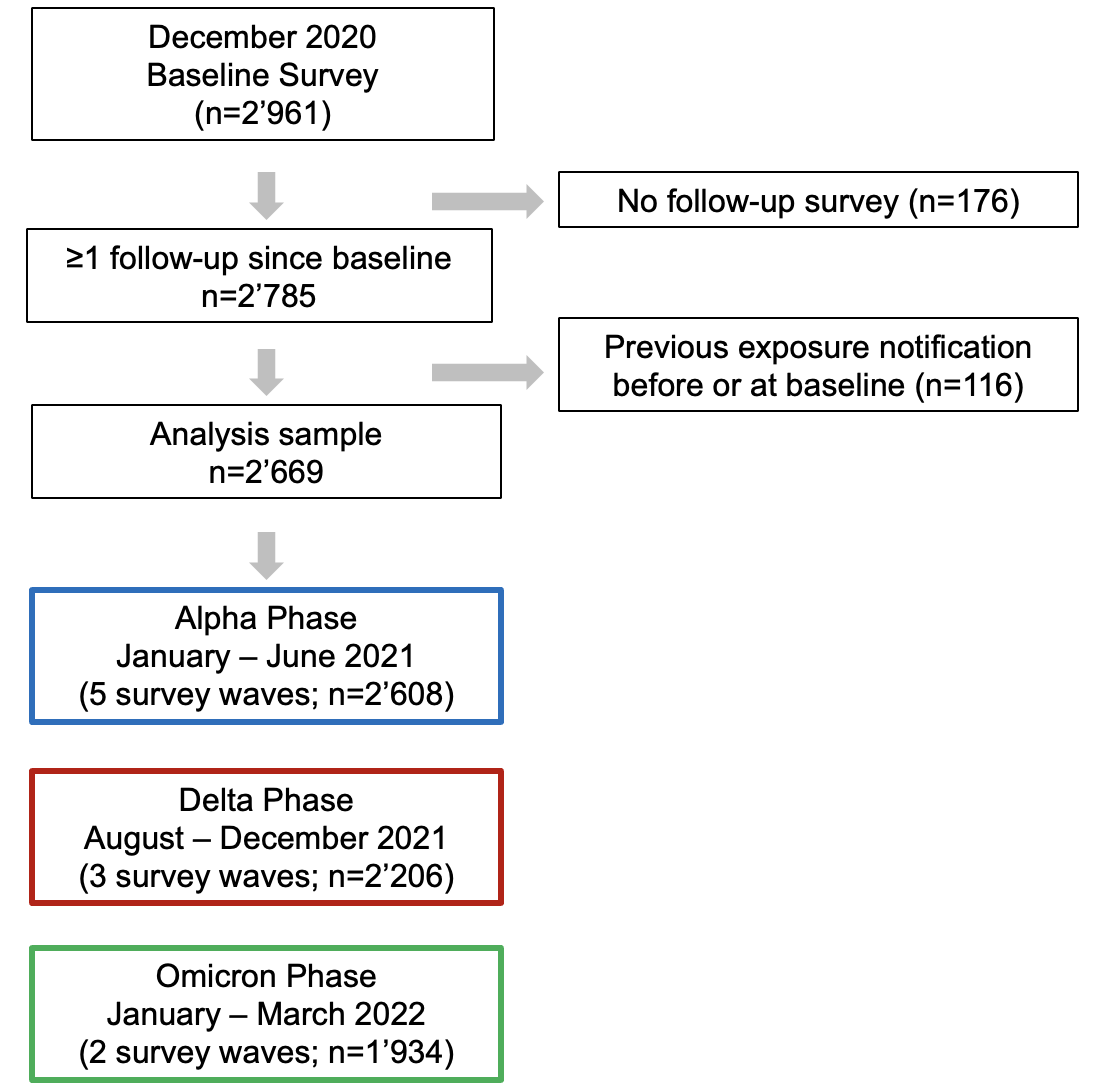


**Supplementary Table 3**: Baseline participant characteristics

|  | **Study sample (n=2669)** |
| --- | --- |
| **Median age [IQR]** | 48 [35;59] |
| **Gender** |  |
| Male | 1367 (51.2%) |
| Female | 1302 (48.8%) |
| **Maximum achieved education** |  |
| Only mandatory school | 158 (5.9%) |
| Secondary school, vocational training | 1269 (47.5%) |
| Higher education | 1242 (46.5%) |
| **Employment status** |  |
| Employed | 1892 (70.9%) |
| Unemployed | 777 (29.1%) |
| **Monthly household income** |  |
| <CHF 6000 | 712 (26.7%) |
| CHF 6000 - 10000 | 867 (32.5%) |
| >CHF 10000 | 635 (23.8%) |
| Household income information not provided | 455 (17.0%) |
| **Nationality** |  |
| Swiss citizenship | 2130 (79.8%) |
| Non-Swiss citizenship | 210 (7.9%) |
| Double citizenship (Swiss and non-Swiss) | 329 (12.3%) |
| **Language region** |  |
| German | 1703 (63.8%) |
| French | 575 (21.5%) |
| Italian | 391 (14.6%) |
