# Supplementary material for: Performance of the Swiss Digital Contact-Tracing App Over Various SARS-CoV-2 Pandemic Waves: Repeated Cross-sectional Analyses
Source: JMIR Public Health Surveill. 2022 Nov 11;8(11):e41004. doi: 10.2196/41004 (PMC9700234; doi:10.2196/41004)
Supplement: Multimedia Appendix 5 [file publichealth_v8i11e41004_app5.docx]

**Multimedia Appendix 5**: Description of Venn diagram and subpopulations

**Supplementary Figure 3**: Venn diagram of cumulative outcomes of interest, as estimated over the full study period. The diagram illustrates the population of all users and SwissCovid app users (values in the square [] brackets) who were tested, tested positive for SARS-CoV-2, and/or entered quarantine or isolation following an exposure notification. Each (non)overlap represents a subpopulation of the survey respondents. The breakdown by pandemic phase is provided in **Supplementary Table 4**.


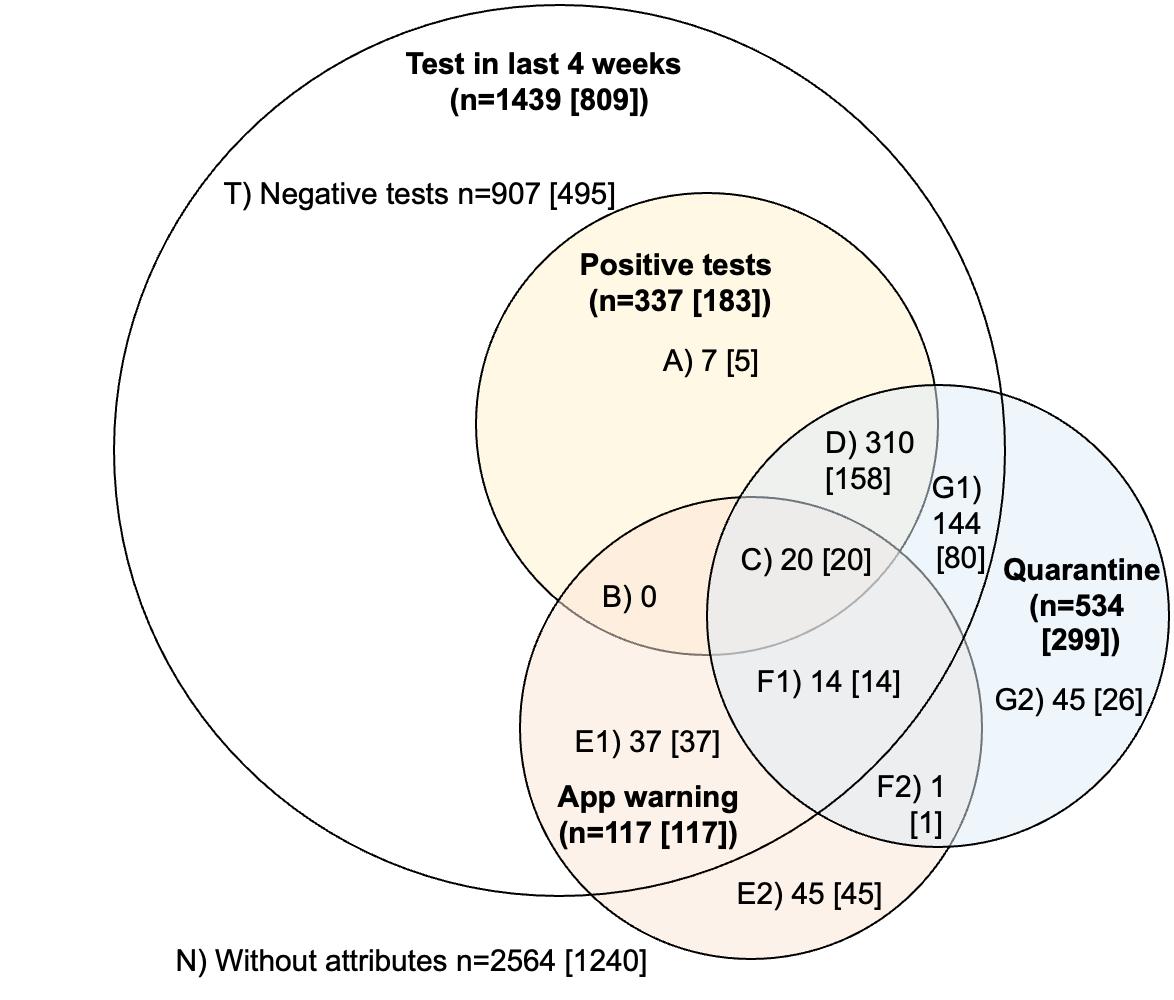


**Supplementary Table 4:** Alternative data aggregations for different Venn diagram segments according to SwissCovid app use status and pandemic phase

|  | **All persons** | **App users only** | | | |
| --- | --- | --- | --- | --- | --- |
| **Venn Segment** | **Full period** | **Full period** | **Alpha** | **Delta** | **Omicron** |
| A | 7 | 5 | 3 | 0 | 2 |
| C | 20 | 20 | 7 | 2 | 11 |
| D | 310 | 158 | 26 | 13 | 119 |
| E.1 | 37 | 37 | 17 | 4 | 16 |
| E.2 | 45 | 45 | 26 | 9 | 10 |
| F.1 | 14 | 14 | 13 | 1 | 0 |
| F.2 | 1 | 1 | 1 | 0 | 0 |
| G.1 | 144 | 80 | 49 | 12 | 19 |
| G.2 | 45 | 26 | 11 | 3 | 12 |
| Without attributes | 2564 | 1240 | n.d. | n.d. | n.d. |
| X | 907 | 495 | n.d. | n.d. | n.d. |
